# Application of the RE-AIM framework to evaluate a stepped care intervention for adolescents and youth living with HIV in Kenya: a mixed methods approach

**DOI:** 10.1186/s43058-026-00888-0

**Published:** 2026-02-23

**Authors:** Nok Chhun, Dorothy I. Mangale, Kawango Agot, Sarah Masyuko, James Kibugi, Wenwen Jiang, Sarah Hicks, Jacinta Badia, Winnie A. Owade, Nancy A. Ounda, Olivia A. Okumu, Lilian A. Ouma, Philip O. Odote, Veronica A. Songa, Pamela K. Kohler, Grace John-Stewart, Kristin Beima-Sofie

**Affiliations:** 1 Department of Global Health, University of Washington, Seattle, WA USA; 2 Department of Oncology, Washington University, St. Louis, MO USA; 3 Impact Research and Development Organization, Kisumu, Kenya; 4 Department of Epidemiology, University of Washington, Seattle, WA USA; 5 Department of Child, Family, and Population Health Nursing, University of Washington, Seattle, WA USA; 6 Department of Pediatrics, University of Washington, Seattle, WA USA; 7 Department of Medicine, University of Washington, Seattle, WA USA

**Keywords:** RE-AIM, Implementation science, Kenya, Adolescent and youth, Stepped care

## Abstract

**Background:**

Recently expanded WHO guidelines on differentiated service delivery (DSD) include expanded eligibility for adolescents and youth living with HIV (AYLHIV). We evaluated implementation of a stepped care program that included DSD for stable AYLHIV and intensified services, including mental health counseling, for AYLHIV with greater needs.

**Methods:**

We used the Reach, Effectiveness, Adoption, Implementation, and Maintenance (RE-AIM) framework to guide evaluation of the Data-informed Stepped Care (DiSC) study, a cluster randomized controlled trial implemented from April 2022 to August 2023 in 24 HIV care facilities in Kenya. We used a mixed methods convergent parallel design to evaluate performance indicators across RE-AIM dimensions. Surveys were analyzed using descriptive statistics and qualitative data using directed content analysis.

**Results:**

Of 3,945 AYLHIV ages 10–24 years old attending care at intervention facilities, 933 AYLHIV were screened and 895 were enrolled, representing an enrollment rate of 96% and 23% *reach* of the intervention. Distribution by age groups were 10–14 years: 29%; 15–19 years: 48%; 20–24 years: 24%. Perceived *effectiveness*, including improved retention and viral suppression among AYLHIV, motivated continued implementation throughout the study duration. Providers also identified opportunities to improve AYLHIV outcomes by highlighting the importance of integrating mental health into HIV care programs. Prior to implementation, 49 health providers were trained to deliver the DiSC intervention, representing *adoption* by 25% of the total facility workforce, including 95% of clinical officers and 56% of nurses. *Implementation* was facilitated by provider-identified, fidelity-consistent adaptations to optimize contextual fit of the intervention. Key determinants influencing *implementation* were provider collective efficacy, compatibility with clinic workflows, leadership engagement, and alignment with changing national guidelines. Post-trial, providers supported continued use of the DiSC intervention (*maintenance),* citing leadership support, training, and material and human resources as key influencers on future sustainment.

**Conclusions:**

Applying RE-AIM to evaluate performance indicators of a stepped care program for AYLHIV identified high adoption and perceived effectiveness, and key influences on implementation and maintenance. Providers were motivated to adopt and sustain use of the DiSC intervention because of perceived positive impact on health system efficiencies and AYLHIV outcomes.

**Trial Registration:**

ClinicalTrials.gov, NCT05007717. Registered 13 July 2021.

Contributions to the literature

- By studying the impact of expanded service delivery strategies that included a focus on mental health alongside revised guidelines for differentiated service delivery (DSD), we generated timely and critical knowledge to support implementation of DSD and mental health support for adolescents and youth living with HIV (AYLHIV).
- We identified that differentiation of HIV care services improves AYLHIV outcomes; providers perceived that AYLHIV are maintaining their adherence, clinic appointments, and viral suppression.
- Our evaluation applies qualitative methods to each dimension of RE-AIM and adds to the limited published literature using this approach.
- Providers found that the stepped care tool guided them in providing high-fidelity HIV care aligned with client needs and preferences, while improving system efficiencies.

## Background

Adolescents and youth ages 10–24 living with HIV (AYLHIV) continue to experience high HIV incidence and HIV-related mortality [1–3]. In sub-Saharan Africa, where 84% of AYLHIV aged 15–19 reside [2], AYLHIV have greater likelihood of disengaging from HIV care than older adults [4, 5]. Evidence-based interventions to improve outcomes among AYLHIV are needed to address the critical gap in reaching viral suppression, with retention in care being crucial for achieving the third UNAIDS 95–95-95 target [6] for this priority population.

Adolescents and youth represent a diverse population, with unique influences on their ability to remain in care. Interventions should be tailored to better meet individual health needs for HIV care and management. Stepped care models have been utilized in healthcare settings to improve health outcomes by matching available services to need [7–9]. This service delivery approach improves health system efficiencies by allocating resources to individuals who need more intensive services to manage their health while maintaining or simplifying care for those who are doing well. In resource-constrained settings experiencing a high burden of youth living with HIV and a health care workforce shortage, stepped care approaches may ensure health systems are not overburdened and could facilitate AYLHIV receiving the support they need to maintain life-long engagement in care. In Kenya, stepped care has already been applied to support young people with information about HIV and sexual reproductive health [7, 10]. A stepped care approach may also be an effective strategy for addressing retention and viral suppression among AYLHIV.

In contrast to more intensified services, differentiated service delivery (DSD) has enabled workforce optimization through allowing those with lower support needs to access health services less frequently. The recent change in World Health Organization (WHO) guidelines [11] expanding DSD eligibility to AYLHIV offers an opportunity to examine the potential for DSD to improve both AYLHIV health outcomes and workforce efficiencies. The Data-informed Stepped Care (DiSC) study was a cluster randomized controlled trial implemented in 24 HIV care facilities in western Kenya from April 2022 to August 2023. DiSC was a health system intervention delivered by health providers who used a clinical assessment tool, along with routinely available data, and a stepped care service delivery approach to place AYLHIV into appropriate levels of care (differentiated care, standard of care, individual counselling, and intensive support services) [12]. Using this stepped care approach, providers aligned intensity and frequency of services with the level of support AYLHIV needed to stay in care and achieve viral suppression. DiSC integrated mental health services into the stepped care delivery model, addressing the critical relationship between AYLHIV mental health and HIV-related outcomes [13–16] as well as the gap in the number of health providers trained to provide mental health services in resource-constrained settings [17]. For example, there are only a median of 0.12 child and adolescent mental health providers per 100,000 population in low-middle-income countries compared to 4.56 per 100,000 reported for high income countries. As part of DiSC, AYLHIV who needed higher levels of support received more intensive services, such as cognitive behavioral therapy and enhanced adherence counseling sessions, psychosocial interventions recommended by the WHO to address mental health needs and improve outcomes for AYLHIV [18]. In contrast, AYLHIV who were enrolled in DiSC and stable benefited from differentiated care elements such as fast-track pharmacy refills and longer visit intervals.

Implementation information is important for understanding how to effectively translate research [19, 20] to practice and ensure sustainment of programs that improve AYLHIV clinical outcomes. Currently, information about adoption, reach, implementation, and integration of DSD into HIV care programs is limited. Using a mixed methods approach, we evaluated implementation of the DiSC stepped care intervention to increase knowledge about whether matching AYLHIV needs to services could simultaneously improve workforce efficiencies alongside health outcomes among this priority population.

## Methods

### HIV care clinic setting and study population

The DiSC study was implemented in 12 intervention HIV care facilities in Kisumu (n = 3), Homabay (n = 4) and Migori (n = 5), all high HIV prevalence counties in western Kenya. Compared to the national prevalence of 4.5% reported for adults aged 15–49 years old, HIV prevalence was 18.5% for Homabay, 17.5% for Kisumu, and 11.8% for Migori counties [21]. Facilities ranged from a Level 2 to Level 4 designation and were classified as dispensaries and clinics, health centers, or sub-county hospitals, respectively [22]. Participating intervention facilities were 83% (10/12) public and 20% (2/12) faith-based organizations. Eligibility criteria for participating facilities included use of electronic medical records (EMR), client volume (≥ 100 AYLHIV ages 10–24 years old enrolled in care), and facility leadership interest in participation [12]. Adolescents and youth were recruited from these facilities during the window of enrollment if they were aged 10–24 years old, in HIV care, and were aware that they were living with HIV.

### The DiSC intervention

**Fig. 1:**
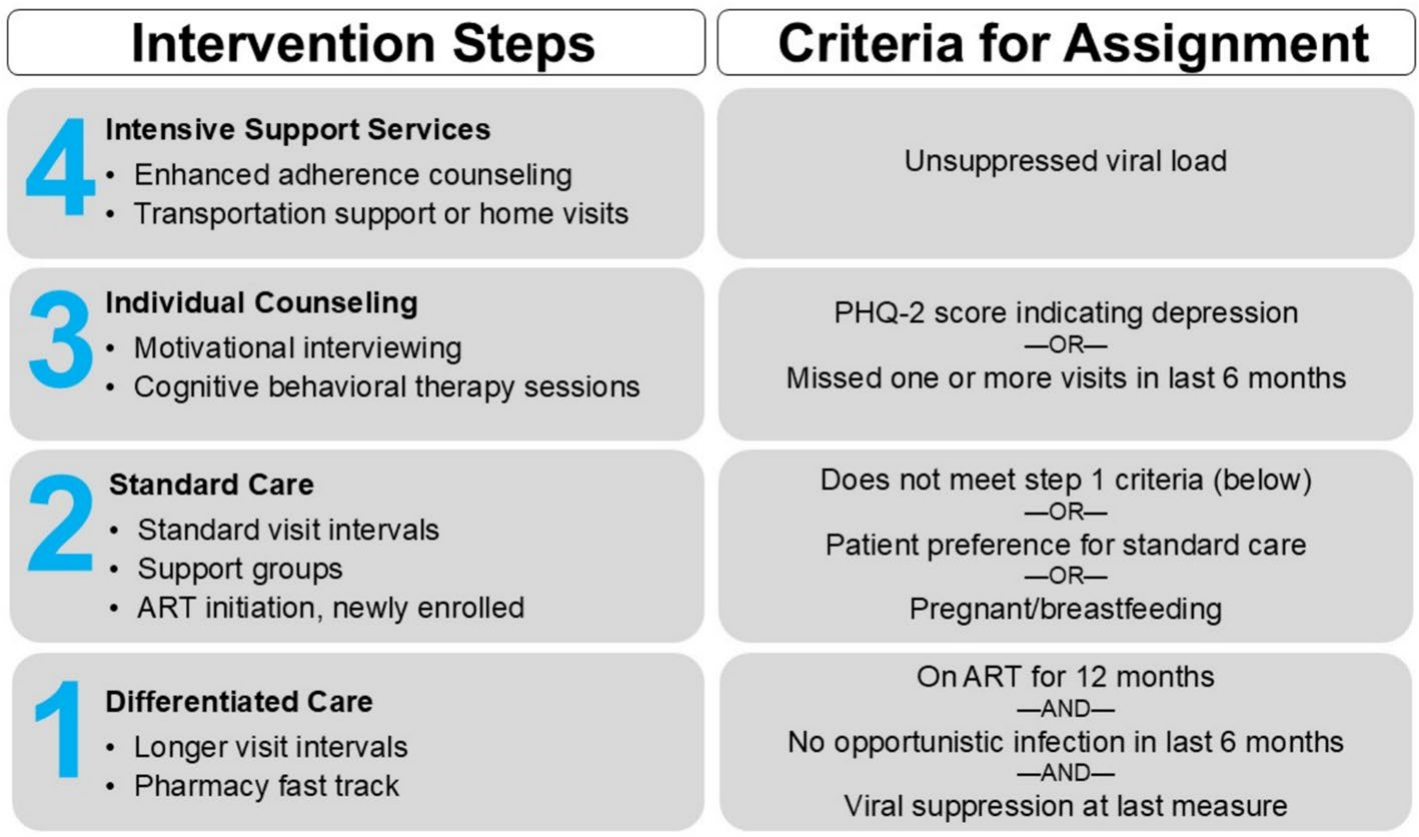
Data-informed stepped care (DiSC) intervention. DiSC is a multi-component intervention that uses routinely available data to match AYLHIV to one of four steps to support retention in care

DiSC was a multi-component intervention testing provision of a stepped care program that included DSD elements and intensified services. As a core component of DiSC, a clinical assessment tool (Fig. 1) was used by health providers to assign AYLHIV to care “steps” which corresponded with evidence-based interventions to support retention in care. Providers used routinely available health record data such as viral load information, length of time on ART or whether AYLHIV missed a visit to allocate them to receive the services assigned to their appropriate step of care. The lowest care step, Step 1, is aligned with differentiated service delivery strategies where HIV services offered to AYLHIV included fast-track pharmacy refills and longer visit intervals. As AYLHIV stepped up, visit frequency and service intensity increased, including provision of mental health counseling and motivational interview sessions (Step 3), and enhanced adherence counseling sessions and case management services such as home visits (Step 4). Youth enrolled from control facilities and AYLHIV allocated to Step 2 received standard of care per Government of Kenya guidelines [23, 24].

### Study design

We used a mixed methods convergent parallel design [25] to evaluate DiSC stepped care intervention implementation, using varied sources of data collected from October 2021 to November 2023. Using this mixed methods approach, we collected quantitative and qualitative data concurrently and compared results for concordance.

### Theoretical framework

We used the Reach, Effectiveness, Adoption, Implementation, and Maintenance (RE-AIM) framework [20] to guide our evaluation of DiSC intervention performance (Table 1). Because of its systematic approach, RE-AIM provides a comprehensive evaluation of health program impact. Within our study, implementation outcome measures were defined as follows: 1) *Reach* – participation and representativeness of the target population for the intervention, 2) *Effectiveness* – actual and perceived impact on health-specific outcomes, 3) *Adoption* – uptake of the intervention by health providers, 4) *Implementation* – consistent delivery of the intervention as intended, adaptations made to optimize delivery, and barriers and facilitators experienced, and 5) *Maintenance* – perceptions of sustainability of the intervention and it’s benefits over time [20, 26]. We expanded the definition of effectiveness to include perceived effectiveness because at the time of data collection, providers were not aware of actual trial outcomes, therefore their responses were based on their perception of intervention effectiveness. Additionally, given the 12-month timeframe of the clinical trial, we were not able to assess longer term maintenance. As a result, we report determinants of future sustainment. We previously reported findings focused on effectiveness [27] and implementation [28, 29] dimensions of RE-AIM; however, we summarize previous findings where relevant to present a fuller description of how RE-AIM was applied to evaluate the *who, what, where, when*, and *how* performance indicators of the DiSC stepped care program across all framework dimensions.

**Table 1:** RE-AIM Evaluation framework dimensions, description, and data sources*

Dimension	Description	Level	Data Sources
**Reach**: Representativeness of the target population (10–24 years old) for the DiSC intervention	**Question:** What was the percent of the target population reached by the intervention? Why did/didn’t someone participate? Was there adequate participation across all age categories (10–14, 15–19, 20–24)? **Evaluation strategy:** Use of qualitative methods to understand reach and engagement with the target population	Client Provider	Facility electronic medical record (EMR) DiSC study case report forms Provider interviews
**Effectiveness: Perceived** effects of the DiSC intervention, both positive and negative, including unintended consequences	**Question:** Was there any intervention impact on perceived effectiveness? **Evaluation strategy:** Use of qualitative methods to understand effects of the DiSC intervention on health-specific outcomes, including unintended consequences	Client Provider	Primary RCT outcomes reported elsewhere Provider interviews
**Adoption**: The number and proportion of providers trained to deliver the DiSC intervention	**Question:** What percentage of facility workforce participated in training on the DiSC intervention? How many were trained on mental health? **Evaluation strategy:** Use of qualitative methods to understand factors influencing adoption of the DiSC intervention	Provider	DiSC study case report forms End-of-trial survey Provider interviews
**Implementation**: Extent to which the DiSC intervention was implemented as intended, adaptations made, and barriers and facilitators	**Question:** What percentage of facilities made adaptations? What were the adaptations made? What were the barriers and facilitators that impacted implementation? **Evaluation strategy:** Use of qualitative methods to understand implementation (What multi-level contextual determinants matter for implementation? What factors influenced consistent delivery?)	Provider	Adaptations and determinants reported elsewhere End-of-trial survey Provider interviews
**Maintenance**: Extent to which the DiSC intervention and its benefits is sustained over time	**Question:** What is the likelihood of continued use of the DiSC intervention by providers? What are key influencers of future sustainment? **Evaluation strategy:** Use of qualitative methods to understand facility-level institutionalization of the DiSC intervention	Provider	End-of-trial survey Provider interviews

^*^Data sources do not include those reported for Effectiveness and Implementation dimensions in other publications

### Data collection

#### Quantitative data

##### Electronic medical record data abstraction

Data to evaluate the representativeness of our target population were abstracted from the electronic medical records (EMR) in October 2021, prior to the start of DiSC trial enrollment in April 2022. Data abstracted from the EMR was used to compare demographic information of those attending care at intervention sites and those that enrolled in the trial. The EMR was also used to abstract data on the primary outcome measure for *effectiveness*, which was retention in care, defined as a missed visit (within a 30-day time period for any visit) and 12-month loss to follow-up [12]. A secondary measure, viral suppression, was abstracted from the Kenya National AIDS & STI Control Program database.

##### Case report forms

Intervention study sites participated in a 3-day training focused on use of the DiSC clinical assessment tool and delivery of corresponding services. Study case report forms were used to assess the number of clinical officers and nurses who participated in DiSC implementation training; a subset of these providers, in addition to adherence counselors at the facility (who were not part of DiSC implementation training) were trained to deliver the mental health components that were part of the higher intensity level services offered during the trial. The mental health training consisted of one week in-person didactic instruction, and virtual mentored counseling and systematic feedback with providers delivering mental health sessions to AYLHIV post training.

##### End-of-trial surveys

The DiSC study is part of the *Prevention and Treatment through a Comprehensive Care Continuum for HIV-affected Adolescents in Resource Constrained Settings* (PATC^3^H) consortium. All health providers from intervention facilities completed surveys that included evaluation of adoption, implementation, and maintenance dimensions of RE-AIM using standardized questions [30] adopted by all PATC^3^H consortium teams to ensure data harmonization across studies in this network [31, 32]. The quantitative survey was administered to 37 providers from July to November 2023 and assessed provider perceptions of the intervention, the training they received, whether providers consistently implemented the DiSC intervention as intended, and the extent to which the DiSC intervention would be sustained over time.

#### Qualitative data

##### Interviews

In-depth interviews (IDIs) were conducted with 2 health providers from each intervention facility in August 2023. Interviews were conducted by a team of experienced Kenyan social scientists (WAO, NAO, OAO, OLA, POO, VAS) using a semi-structured discussion guide informed by the Consolidated Framework for Implementation Research [33] and refined (NC, DIM, KBS) to cover the following topics: (1) provider experience delivering the DiSC intervention, both the clinical assessment tool and services; (2) provider perception of intervention adaptability during implementation; (3) perceived impact of the intervention on health-related outcomes; and (4) likelihood of continued use of the DiSC clinical assessment tool and perception of resources needed to ensure sustainability. Providers were ≥ 18 years old, clinical officers or nurses, and were purposely selected for participation in the IDIs to reflect a range of experiences during implementation. Providers were eligible if they were trained on DiSC, used the clinical assessment tool to assign AYLHIV to available services at their intervention site, and were interested in participating. Sample size was determined based on information power [34]. IDIs were conducted in English, but providers were also encouraged to use Kiswahili if preferred. Discussions lasted a median of 67 min (interquartile range [IQR]: 58, 75), and were audio-recorded, translated as needed, and transcribed verbatim (WAO).

#### Data analysis

##### Quantitative data analysis

Descriptive statistics were used to summarize counts, proportions, means, standard deviations, medians, and interquartile ranges. To assess *reach* of the total clinic population, we defined the denominator for our target population as the total number of AYLHIV ages 10–24 years old attending care at intervention facilities. *Adoption* was calculated as the proportion of clinical officers and nurses trained to deliver the DiSC intervention, out of the total number of health providers in these cadres, as well as the overall number of healthcare workforce at the facilities.

End-of trial surveys assessing provider perceptions of quantitative measures of adoption, implementation, and maintenance dimensions were measured on a Likert scale and visually displayed using diverging bar charts. All quantitative analyses were performed using the R statistical computing environment [35].

##### Qualitative data analysis

Health provider interview data were analyzed using a directed content analysis approach [36], and an initial codebook was developed (NC). The codebook was organized around the RE-AIM dimensions with specific code constructs within each dimension [37]. The codebook was further refined through group discussion (NC, DIM, KBS). Two team members (NC, DIM) tested the codebook by coding a common transcript and resolved differences in code application through discussion until consensus was reached. The remaining transcripts were divided for independent coding by one member of the team, code application evaluated by another team member, and discrepancies discussed to achieve group consensus (NC, DIM). Queries and code co-occurrence tables were generated to summarize findings within RE-AIM dimensions. *ATLAS.ti* software (version 24) was used to facilitate data management and analysis.

##### Mixed methods analysis

Qualitative and quantitative approaches were used to achieve triangulation, convergence, and expansion of findings. Quantitative data were summarized and qualitative queries were then generated to evaluate the rationale underlying the quantitative findings. The qualitative data were further searched for additional information not presented yet, which was then compared to the quantitative results to provide an integrated summary of overall findings.

## Results

Twenty-four in-person interviews were conducted with health providers. The majority of providers were clinical officers (74%), identified as female (52%), and had a median age of 35 years (IQR: 32, 41). Providers reported a median 5 years (IQR: 3, 8) at their current clinic, and a median of 8 years (IQR: 6, 12) engaged in AYLHIV care and treatment. Respondent characteristics of providers who participated in the post-trial survey were similar (Table 2).

**Table 2:** Demographic characteristics of health providers across DiSC trial intervention facilities

	Survey (N = 37)	Interview (N = 23)*
**Characteristic**	***N* (%) or** **Median [IQR]**	***N* (%) or** **Median [IQR]**
Primary work location		
Comprehensive care clinic	27 (73)	18 (78)
Other^1^	10 (27)	5 (22)
Gender		
Male	14 (38)	11 (48)
Female	23 (62)	12 (52)
Age (years)	34 [31, 39]	35 [32, 41]
Highest level of education: university/college	37 (100)	23 (100)
Healthcare provider classification		
Clinical officer	25 (68)	17 (74)
Nurse counselor	4 (11)	3 (13)
Nurse	8 (22)	3 (13)
No. of years at current clinic	5 [3, 8]	5 [3, 8]
No. of years providing HIV care to PLHIV (all ages)	8 [6, 10]	8 [6, 13]
No. of years providing HIV care to AYLHIV (ages 10–24 years)	8 [6, 10]	8 [6, 12]

*Abbreviations*: *AYLHIV *adolescents and youth living with HIV, *PLHIV *People living with HIV

^*^Information for five providers were abstracted from a demographic survey administered prior to the start of DiSC trial implementation, one provider missing demographic information and not included in this table

^1^Other category includes prevention of vertical transmission, antenatal care, maternal child health, and outpatient departments. Due to rounding percentages may not equal 100 percent

### Reach

A total of 3945 AYLHIV ages 10–24 attended routine HIV care at intervention facilities, of whom, 933 were screened and eligible, and 895 were enrolled in the clinical trial [38]. Reach was estimated to be 23% (895/3945) of the total clinic population. Within the context of a clinical trial, we report an enrollment rate of 96%, which is the proportion of AYLHIV enrolled among those screened. When enrollment was evaluated by specific age groups, age distribution was 29% (255/895) for AYLHIV 10–14 years, 48% (426/895) for AYLHIV 15–19 years, and 24% (214/895) for AYLHIV 20–24 years; indicating that our enrollment was highest among 15–19 years old and thus not representative of the overall higher clinic distribution of AYLHIV 20–24 years attending care. Among those enrolled at the intervention facilities, gender distribution was 39% male and 59% female, which reflected the overall distribution of those attending care (35% male vs. 65% female). However, distribution varied when stratified by different age categories (Table 3).

**Table 3:** Age and gender distribution of adolescents and young adults living with HIV across DiSC trial intervention facilities

	**All Clients at Intervention Facilities**			**Participants Enrolled in DiSC Trial**		
Age categories	Overall (N = 3945)	Female (N = 2581)	Male (N = 1364)	Overall (N = 895)	Female* (N = 532)	Male* (N = 353)
Overall	3945	2581 (65.4%)	1364 (34.6%)	895	532 (59.4%)	353 (39.4%)
10–14	1165 (29.5%)	601 (23.3%)	564 (41.3%)	255 (28.5%)	132 (24.8%)	119 (33.7%)
15–19	1178 (29.9%)	692 (26.8%)	486 (35.6%)	426 (47.6%)	233 (43.8%)	194 (55.0%)
20–24	1602 (40.6%)	1288 (49.9%)	314 (23.0%)	214 (23.9%)	167 (31.4%)	40 (11.3%)

^*^2 participants reported transgender and 8 participants missing gender in the behavioral survey at enrollment

When discussing representativeness of enrolled AYLHIV, providers acknowledged that the DiSC intervention was part of a research study with an enrollment target, which limited the number of AYLHIV able to participate from each site.

*“[T]he challenge was that, during the enrollment, when we were doing the study, we were given a maximum number to reach in enrolling the client. So some of our clients missed, because we have a bigger number, a bigger [number of] adolescents. But I think we were given up to around 50, something like that. And some of them missed the study. And to us we thought that all of them would benefit during this time. That is one of the challenges we had. That one disadvantaged some of our clients.”* – [P2, Facility 3]

Reaching AYLHIV aged 10–14 years required additional coordination by providers to ensure involvement of parents/caregivers. Providers described how Kenyan parental/caregiver consent guidelines for research participation for AYLHIV < 18 years made it challenging to enroll younger AYLHIV, even though this population is accurately represented.

*“[W]hen we were starting to implement the tool. We needed to have a guardian sign that they have agreed that the adolescent should participate in this study. So, you find some guardians, they don't accompany their children to the hospital. So, you cannot start stepping such a kid if a consent form has not been signed. It has been a challenge at some point.”* – [P3, Facility 1]

While the DSD service delivery model of 3-month visit intervals appealed to younger AYLHIV, providers perceived that already existing eligibility for differentiated services with a longer 6-month visit interval negatively affected willingness to enroll in DiSC among older AYLHIV (ages 20—24), as evidenced by the lower reach in this age group.

*“… for the 20 to 24, sometimes we now book them… they have to only come to clinic twice in a year so in between they can be coming for the refill. But for the tool… the maximum that you can give them is now that three months… they'll tell you, "Doctor, the three months will inconvenience me. I'm working in Nairobi. So if you give me that, the transport is not there.”* – [P1, Facility 5]

### Effectiveness

Clinical trial effectiveness outcomes have been previously reported [27]. In summary, a total of 1911 AYLHIV were enrolled from intervention and control facilities. At intervention facilities using the stepped care tool, 574 (65%) AYLHIV were assigned to differentiated services, 122 (14%) to standard care, 100 (11%) to mental health and retention counseling, and 92 (10%) to intensive support services. Most AYLHIV were retained in care, attending 92% of scheduled clinic appointments. There were no significant differences between intervention and control facilities in the number of missed visits (8.5% vs. 8.3%) or viral non-suppression (7.7% vs. 9.7% respectively) [27]. Differentiated elements, i.e., pharmacy fast-track and longer visit intervals were significantly different between intervention and control facilities; fast-track pharmacy visits increased while the number of scheduled visits decreased at intervention facilities [27].

Despite absence of impact on retention and viral suppression outcomes, at the time of the interviews and in the absence of clinical trial outcome data, providers perceived that the intervention was effective in supporting AYLHIV with retention in care, viral suppression, and adherence. Providers felt this benefit was especially helpful for AYLHIV who received enhanced services offered in response to viral non-suppression or mental health screening.

*“Suppression has been good. Like I'm saying it's 99% as at now. It has really enabled us to categorize our mental health participants into their clinics and cater for their needs.”* – [P3, Facility 12]

Providers recognized the burden of mental health and described how AYLHIV poor health outcomes are related to their mental health status, with recognition of how both disclosure and transition impact mental health outcomes.

*“[B]y this tool bringing that tool where you can screen for depression; it was a major milestone because …these adolescents face so many issues, yes both at home, at school and also during the transitions when they are transitioning from child to adolescent then to adulthood. So the issue of mental health when it came with this tool, it helped a lot. Because you find even the issues how disclosure is done, the issue of when an adolescent wants to transition to a boarding school. So these things were affecting their adherence and it’s because of that issue of the mental part; the mental health issue. So, by this form bringing that part it was a good step, yes, towards also helping these adolescents who have mental issues.”* – [P2, Facility 1]

Another positive outcome providers noted was improvement in overall provider–client relationships, describing more openness from AYLHIV during clinic visits.

*“They feel comfortable in our clinics, they feel that at least they're served well in our clinics. So, when you take time with them, talking to them using the DiSC tool, asking them questions, they feel like they're loved. They feel so protected in our clinic, they feel like the next time they want to come back, you know, so it helped us in retaining them. And also, management of the high viral loads, those with high viral loads. It helped us to follow them closely, giving them shorter TCAs [clinic appointments], counselling them, so it also helped us.”* – [P3, Facility 8]

### Adoption

Prior to implementation, 49 health providers across participating intervention facilities were trained to deliver the DiSC intervention. This represented 25% (49/199) of the total health facility workforce, involved in adolescent HIV services, 95% of facility-based clinical officers (35/37), and 56% of facility-based nurses (14/25). Additionally, nine clinical officers, two nurses, and 22 adherence counselors (total n = 33) were trained to deliver the cognitive behavioral therapy sessions for AYLHIV assigned to higher intensity level services; 88% completing training (n = 29), and 20 (61%) completing the three required supervision sessions post-training. Due to study oversight during the clinical trial, all facilities implemented the intervention, we did not have non-adopting or partially adopting sites.

**Fig. 2:**
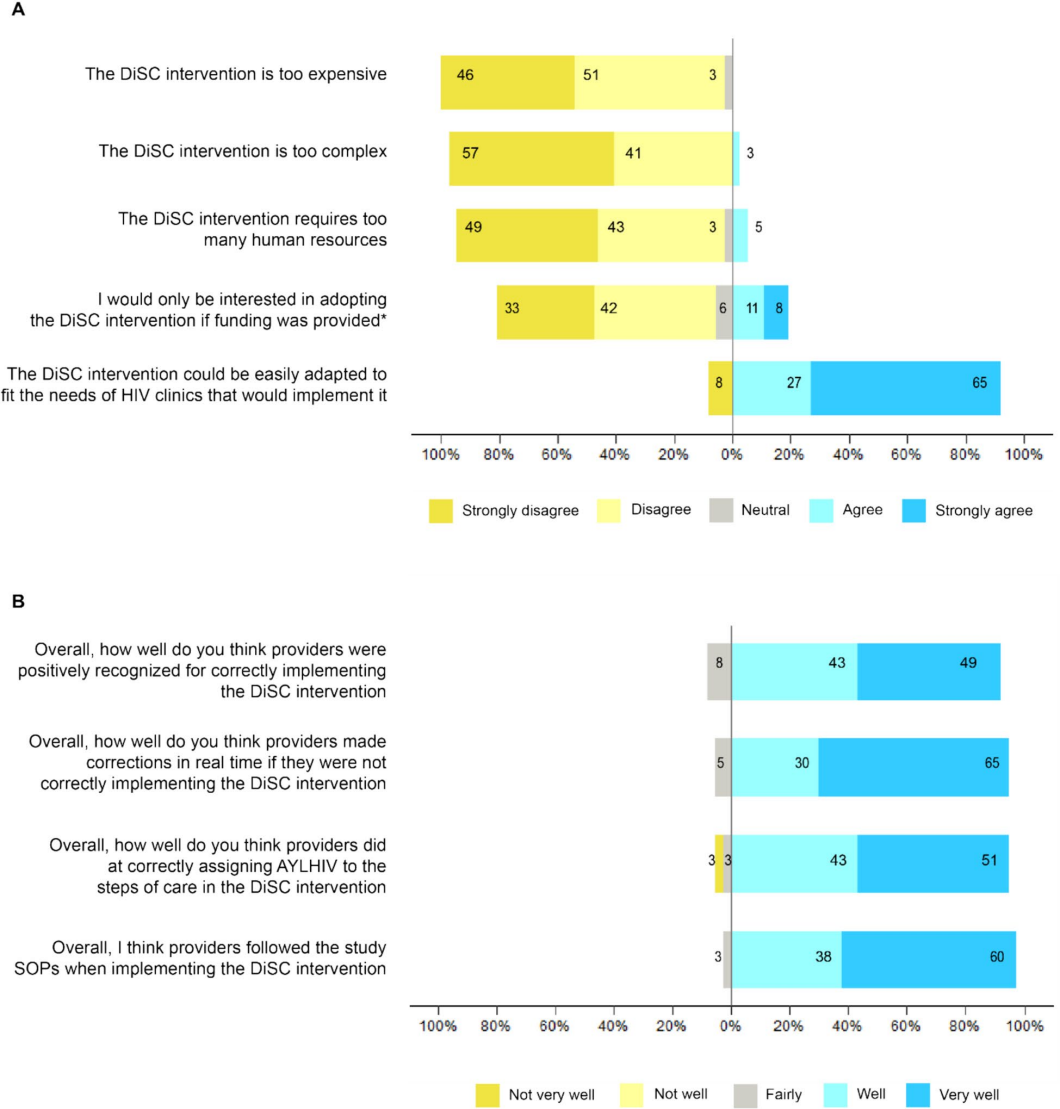
Provider level of agreement with the following statements about factors influencing (**A**) *adoption* measured on a Likert scale of 1 to 5 (with 1 = strong disagreement and 5 = strong agreement) and (**B**) *implementation* measured on a Likert scale of 1 to 5 (with 1 = not very well and 5 = very well), collected from the end-of-trial evaluation survey. Total n = 37 unless otherwise noted, *n = 36. Percentages may not add up to 100 because of rounding. AYLHIV = adolescents and youth living with HIV, SOPs = standard operating procedures

The end-of-trial survey highlighted providers’ perception that the DiSC intervention was easily adaptable to fit the needs of clinics who may implement it (92% agree/strongly agree; Fig. 2A). In response to the statement that the DiSC intervention was too complex, too expensive, or would require too many human resources to implement, 98%, 97%, and 92% disagreed/strongly disagreed respectively. Qualitatively, providers supported their survey responses noting that the clinical assessment tool allowed them to deliver services targeted to AYLHIV needs while decreasing their workload at the same time. Ability to align services to meet AYLHIV needs while improving service efficiencies was a major factor influencing adoption of DiSC.

*“I would first like to appreciate the introduction of the DiSC tool actually, because through the DiSC tool, we managed to provide services to our adolescents as individuals. It really helped us even identify our gaps as healthcare workers, because we were now able to approach the needs of those adolescents as individuals and not as a group. Because initially, we would just brush over some issues without thinking further, even without knowing the specific needs of some of the adolescents who were in the study. So, through the DiSC tool, I can say we've managed to provide appropriate care to our adolescents.”*

– [P3, Facility 2]

*“[W]ith this tool we are able to give longer TCAs [clinic appointments] for the children. So, you'll find maybe an adolescent who is maybe in the university and is going for a longer way, at least if they're stable and if they're suppressed and they have no issues, I'm able to give a longer TCA. Then it has also reduced our workload in that instead of having all the adolescents and just coming for drug refill, we give a longer TCA to the ones who are stable…established and then we remain with the few who might need more care and more time to handle them.”* – [P1, Facility 7]

### Implementation

Adaptations providers made during early phase implementation (May – December 2022) to optimize uptake and delivery of the DiSC intervention have been previously described [28]. In summary, the Framework for Reporting Adaptations and Modifications Expanded [39] was applied to characterize planned adaptations, which providers identified during routinely conducted continuous quality improvement meetings. The majority of adaptations were made to improve integration into routine clinic practices, such as improving documentation, clinic workflows, and addressing scheduling challenges. Primary reasons for adaptations were to align delivery with AYLHIV needs and preferences and address barriers to service access unique to AYLHIV, such as boarding school. All adaptations to optimize DiSC implementation were determined to be fidelity-consistent because none impacted core elements of the intervention, which was step assignment and step reassessment at subsequent visits. Providers also quantified their perceptions of DiSC intervention acceptability, appropriateness, and feasibility using a Likert scale from 1 to 5 (1 = strong disagreement; 5 = strong agreement). Provider perceptions of these three measures were consistently high but did significantly improve over time [40].

Key barriers and facilitators experienced during early phase implementation have also been previously reported in a qualitative assessment [29]. In summary, the Consolidated Framework for Implementation Research (CFIR) was used to examine key determinants influencing implementation. Factors impacting implementation were predominantly from the inner setting, outer setting, and intervention characteristic CFIR domains. Provider collective efficacy was important in consistent implementation of DiSC and was facilitated by the previously described continuous quality improvement meetings, access to knowledge and information as a result of the training they received, and perceived intervention effectiveness. Providers highlighted that leadership involvement was an important driver of implementation success; leadership examples included availing space for mental health counseling sessions and time for client-provider interactions outside standard operating hours.

In the end-of-trial survey, providers perception that the DiSC intervention was implemented with high fidelity persisted. Although 51% of providers (19/37) believed that health providers were similarly motivated at the beginning and end of the intervention period, 30% (11/37) believed that providers were more motivated at the beginning of the intervention. Providers reported that their motivation for continued, consistent use was partially driven by the positive impact the tool had on their work environment.

*“When we're using it [DiSC tool], we're not using it for the sake of only the client. We're using it for the sake of both of us. Because it has helped us in the management of our young adolescent[s], in that we do the right stepping. We do give the right things when they're supposed to be given those. And what can I add? I feel that is enough.”* – [P6, Facility 10]

When asked to rate their preparedness, on a scale of 1 to 10, to deliver the DiSC intervention, providers perceived that the training was adequate (median = 10, IQR: 9, 10). During interviews, providers highlighted the importance of training if the intervention were to be expanded to other facilities.

*“[T]hey have to be trained on the DiSC, so that they understand the tool. The staff or the people who will be taking care of the client, first of all, must understand the tool. They are the people who will be able to talk to the client, to register or to enroll to the tool……And they need to appreciate the goodness of the tool, because the tool is definitely designed to make us improve on our indicators.”* – [P2, Facility 3]

The majority of providers agreed that they followed study standard operating procedures (98% well/very well; Fig. 2B). In addition, their level of agreement with the statements about whether they were assigning AYLHIV to the correct step (94% well/very well), and if not whether they were able to course correct in real time (95% well/very well), demonstrated high collective efficacy during implementation.

*“We, the CQI team, the continuous quality improvement team, if you see that these interventions are not working, then the team will sit down and there's something called the brainstorming. So, for brainstorming, then people will come with the different opinions and we see which one can work. If it doesn't work, we'll take the gaps to the CQI team, and the team will sit down, I think they will come with the change ideas which can work.”* – [P6, Facility 9]

### Maintenance

**Fig. 3:**
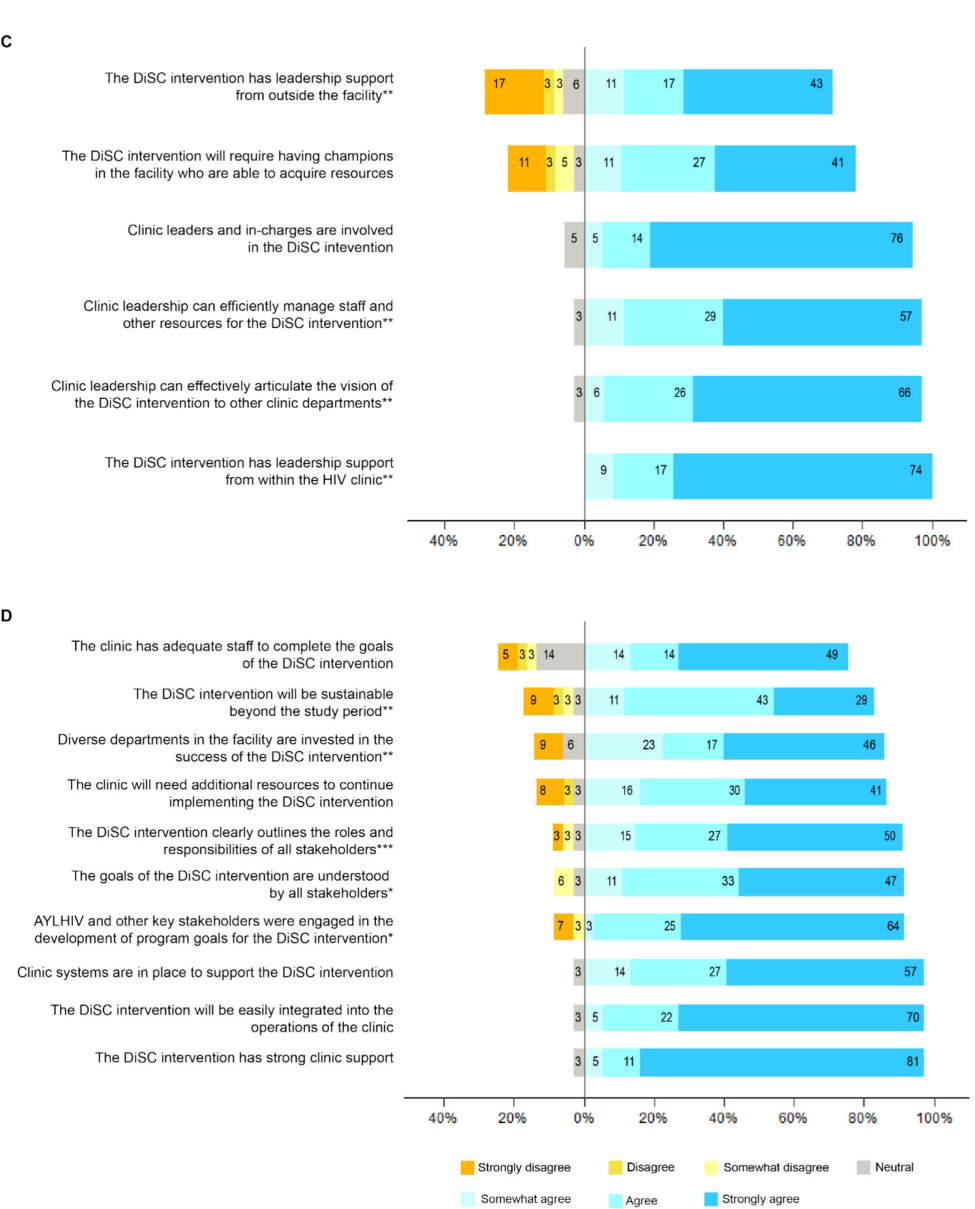
Provider level of agreement with the following statements about factors influencing *maintenance* (**C** and **D**), measured on a Likert scale of 1 to 7 (with 1 = strong disagreement and 5 = strong agreement) collected from the end-of-trial evaluation survey. Total n = 37 unless otherwise noted, *n = 36, **n = 35, ***n = 34. Percentages may not add up to 100 because of rounding. AYLHIV = adolescents and youth living with HIV

Providers perceived that leadership engagement was high and they supported implementation of DiSC (91% agree/strongly agree; Fig. 3C). Additionally, providers agreed that leadership were involved with the DiSC intervention (90% agree/strongly agree) and efficiently managed staff and other resources to support implementation (86% agree/strongly agree). Most providers agreed that the DiSC intervention will be sustainable beyond the study period (72% agree/strongly agree; Fig. 3D) and believed there was strong clinic support for the intervention (92% agree/strongly agree), which could be easily integrated into clinic operations (92% agree/strongly agree). Providers identified potential barriers to sustaining the intervention that were related to material and human resource availability.

*“[T]he key thing would be personnel. Yeah, because without the personnel, it can't kick off. And also the facilities, making sure that the offices are friendly, and the confidentiality of the same, and also just the way I said it that they should be adolescent friendly clinics where they don't feel stigmatized. Much more training, of course, needs to be done on the DiSC tool more so when there's changes, here and there. There need to be [refresher] trainings. Yes. And the other resources, of course, would be the tools and the requirements that are needed, the stepping tool, the DiSC tool, and all those. Those ones are the most important resources that a facility need to consider before having the intervention*.” – [P2, Facility 11]

Providers believed they would continue using the tool because of perceived effectiveness and improved guidance on how to optimize AYLHIV care.

*“I think using the tool is something we can continue with, because we really saw how it impacted on the lives of our adolescents and even me as a clinician, I'm able to feel the weight it took in terms of offering services, and also making it easy for me as a clinician to be able to know what do I need to offer to this adolescent for them to be able to maybe achieve suppression, address their mental health barriers, and also just change the overall thought process of our adolescents.”* – [P1, Facility 8]

To support continued use of the tool, providers recommended a process for continued updates to ensure evaluation criteria in the stepping tool align with changing national guidelines, highlighting the importance of continued adaptation to optimize tool relevance to contextual setting.

*“Changes, I think we said earlier, maybe some indicators need to align to the new guideline…then nowadays we are doing viral load at three months. So, Step 3 [higher intensity services], I said should read enrolled within the last three months. And Step 1 [differentiated services] on ART for more than 12 months, so it should read on ART for more than six months because at six months… we categorize them as stable and unstable at six months.”* – [P1, Facility 6]

Finally, providers felt that sustained use of the DiSC intervention would require leadership support at the national level. Providers recognized policies and policymakers as key influences on standardizing health practices at the facility level, as well as streamlining trainings on DiSC tool use and mental health.

*“The good thing in Kenya, usually the policies are usually standard, they are standardized when it comes from NASCOP, the National AIDS Control Program. It is mandated… So, at policy making level, if you are able convince NASCOP and also if us, we can be given a chance to tell our experiences about the tools because we participated in the study, it can be very good…….But I don’t think you’ll face resistance because the tool was simple, very straightforward”* – [P2, Facility 1]

## Discussion

Using a mixed methods approach, we evaluated the DiSC stepped care intervention to better understand how matching AYLHIV needs to service delivery using a standardized assessment tool could improve workforce efficiencies and AYLHIV health outcomes. Triangulating qualitative and quantitative data enhanced our understanding of DiSC performance indicators along all five dimensions of the RE-AIM framework. We found that providers were motivated to adopt and sustain use of the DiSC intervention because they perceived a positive impact on AYLHIV outcomes and optimization of their time and effort. Additionally, providers felt that integrating mental health into HIV care programs may support improved outcomes in this population by providing support during challenging times, such as disclosure and transition.

Adolescents and young adults represent a diverse group, with varied characteristics such as age, gender, sexual orientation, marital status, as well as how and when they acquired HIV. As a result, their access to care as well as the barriers experienced are similarly varied. It is not surprising that for those in boarding school, it was important that the intervention was adaptable to align their clinic visit intervals with the school calendar. Younger adolescents face barriers to participation due to age of consent laws. Without caregiver consent, adolescents are not able to participate in research, and their lack of representation may limit the development of interventions [41–44]. This finding is consistent with prior studies that identified legal requirements for caregiver consent as a barrier to AYLHIV accessing quality health services. Recommendations to overcome this barrier include family-based strategies [45, 46] that support improved adolescent-caregiver communication, especially related to HIV disclosure. Individuals who know their status are more likely to receive HIV treatment [47] and participate in HIV-related research [48].

DiSC is a health systems intervention delivered by health providers using routinely available data to deliver services. Providers perceived that the intervention improved their client’s health outcomes and noted that the DiSC tool focused their time and resources on AYLHIV who needed more intensive support, thereby improving health system efficiencies. This demonstrates that differentiation of services reallocates resources to where they are most needed, improving efficiencies of health systems [49, 50]. In a study that modified clinic flow procedures for stable clients to allow for longer visit intervals, waiting times improved for clients, and providers had shorter clinic hours which allowed them time to pursue other activities [49].

In order to ensure that the DiSC tool remains relevant, additional adaptations may be needed, such as the flexibility to align with changes in national guidelines. Also, as AYLHIV become candidates for other models of differentiated care, such as community-based ART refills, or qualify for long-acting injectables, the tool will need to be revised to align with these changes. Interventions that can respond dynamically to real-world contexts are more likely to be sustained [51].

Our study had several strengths. We utilized both quantitative and qualitative data to explore each dimension of RE-AIM. Use of qualitative methods helped contextualize quantitative findings to describe not only *what* provider’s thought about the DiSC tool, but *why* they supported implementation and *how* implementation might be adapted to promote sustained use. Our evaluation adds to the limited published literature that applies qualitative methods to each dimension of RE-AIM.

Our analysis has limitations. As a result of having an enrollment target, reach does not represent a meaningful programmatic or clinical estimate. We did not collect individual-level characteristics other than age and gender of AYLHIV from the total population of those attending care. As a result, we cannot assess whether those who participated were representative of those who did not participate. We summarized the effectiveness and implementation dimensions from our previously published work. Furthermore, this evaluation was assessed from the perspectives of health providers who delivered the intervention, with in-depth interviews conducted prior to analysis of the effectiveness outcomes. Therefore, providers supported continued use of the DiSC intervention based on perceived effectiveness, and responses may have differed if the primary effectiveness outcome was available. Additionally, this report does not include AYLHIV experience with the intervention or share their perspectives.

Future studies should evaluate sustainability post-trial and provide practical steps for scale-up throughout Kenya. Prioritization of strategies should focus on ensuring representation from diverse community members to ensure that implementation is acceptable, feasible, and appropriate for new contextual settings.

## Conclusions

Applying RE-AIM to evaluate performance indicators of a stepped care program for AYLHIV identified high adoption and perceived effectiveness, and key influences on implementation and maintenance. We found that having a tool that used routinely available data to match AYLHIV needs to services did simultaneously improve workforce efficiencies and improve their health outcomes. Future studies could explore strategies to improve reach among all 10–24-year-old AYLHIV attending care as well as strategies that build provider capacity to deliver youth-centered interventions. As other models of differentiated care are made available to AYLHIV, use of RE-AIM during planning and evaluation can inform how to increase reach, adoption, and integration of programs that may benefit AYLHIV.

## Data Availability

Quantitative and qualitative data are available upon reasonable request.

## Abbreviations

AYLHIV: Adolescents and young adults living with HIV
CFIR: Consolidated Framework for Implementation Research
CQI: Continuous Quality Improvement
DiSC: Data-informed Stepped Care
DSD: Differentiated Service Delivery
EMR: Electronic Medical Record
FGD: Focus Group Discussion
IDI: In-depth Interview
IQR: Interquartile Range
PATC^3^H: Prevention and Treatment through a Comprehensive Care Continuum for HIV-affected Adolescents in Resource Constrained Settings
PLHIV: People living with HIV
RCT: Randomized Controlled Trial
RE-AIM: Reach, Effectiveness, Adoption, Implementation, and Maintenance framework
SOP: Standard Operating Procedure
TCAs: To Come Again (Clinic appointments)

## References

[1] UNAIDS. Women and HIV. A spotlight on adolescent girls and young women. Geneva, Switzerland: UNAIDS; 2019.

[2] UNICEF. Adolescent HIV prevention. New York: UNICEF; 2024.

[3] The urgency of now. AIDS at a crossroads. Geneva: Joint United Nations Programme on HIV/AIDS; 2024.

[4] Murray KR, Dulli LS, Ridgeway K, Dal Santo L, de Darrow Mora D, Olsen P, et al. Improving retention in HIV care among adolescents and adults in low- and middle-income countries: a systematic review of the literature. PLoS ONE. 2017;12(9):e0184879. 28961253 10.1371/journal.pone.0184879 PMC5621671

[5] Ridgeway K, Dulli LS, Murray KR, Silverstein H, Dal Santo L, Olsen P, et al. Interventions to improve antiretroviral therapy adherence among adolescents in low- and middle-income countries: A systematic review of the literature. PLoS ONE. 2018;13(1):e0189770. 29293523 10.1371/journal.pone.0189770 PMC5749726

[6] UNAIDS. Understanding measures of progress towards the 95–95–95 HIV testing, treatment and viral suppression targets. Geneva, Switzerland: UNAIDS; 2024.

[7] One2One Kenya—Stepped Care model. (2019–2023). Aidsfonds.org. https://aidsfonds.org/project/one2one-stepped-care-model/. Accessed 24 Jul 2024.

[8] Berger M, Fernando S, Churchill A, Cornish P, Henderson J, Shah J, et al. Scoping review of stepped care interventions for mental health and substance use service delivery to youth and young adults. Early Interv Psychiatry. 2022;16(4):327–41. 34018335 10.1111/eip.13180 PMC9292436

[9] van Straten A, Hill J, Richards DA, Cuijpers P. Stepped care treatment delivery for depression: a systematic review and meta-analysis. Psychol Med. 2015;45(2):231–46. 25065653 10.1017/S0033291714000701

[10] Integrating psychosocial interventions and support into HIV services for adolescents and young adults: technical brief. Geneva: World Health Organization; 2023.

[11] World Health Organization. Consolidated guidelines on HIV prevention, testing, treatment, service delivery and monitoring: recommendations for a public health approach. Geneva: World Health Organization; 2021. Licence: CC BY-NC-SA 3.0 IGO. 34370423

[12] Kohler P, Agot K, Njuguna IN, Dyer J, Badia J, Jiang W, et al. Data-informed stepped care to improve youth engagement in HIV care in Kenya: a protocol for a cluster randomised trial of a health service intervention. BMJ Open. 2022;12(10):e062134. 36316073 10.1136/bmjopen-2022-062134 PMC9628651

[13] Bhana A, Mellins CA, Petersen I, Alicea S, Myeza N, Holst H, et al. The VUKA family program: piloting a family-based psychosocial intervention to promote health and mental health among HIV infected early adolescents in South Africa. AIDS Care. 2014;26(1):1–11. 23767772 10.1080/09540121.2013.806770 PMC3838445

[14] Sibinga EMS, Webb L, Perin J, Tepper V, Kerrigan D, Grieb S, et al. Mindfulness instruction for medication adherence among adolescents and young adults living with HIV: a randomized controlled trial. AIDS Care. 2022;34(12):1619–27. 35914112 10.1080/09540121.2022.2105796 PMC9712224

[15] Simms V, Bernays S, Chibanda D, Chinoda S, Mutsinze A, Beji-Chauke R, et al. Risk factors for HIV virological non-suppression among adolescents with common mental disorder symptoms in Zimbabwe: a cross-sectional study. J Int AIDS Soc. 2021;24(8):e25773. 34402199 10.1002/jia2.25773 PMC8368838

[16] Webb L, Perry-Parrish C, Ellen J, Sibinga E. Mindfulness instruction for HIV-infected youth: a randomized controlled trial. AIDS Care. 2018;30(6):688–95. 29067834 10.1080/09540121.2017.1394434 PMC5987527

[17] World Health Organization. Mental Health Atlas 2024. Geneva: World Health Organization; 2025. Licence: CC BY-NC-SA 3.0 IGO. Available at https://www.who.int/publications/i/item/9789240114487.

[18] World Health Organization. Integrating psychosocial interventions and support into HIV services for adolescents and young adults: technical brief. Geneva: World Health Organization; 2023.

[19] Glasgow RE, Estabrooks PE. Pragmatic applications of RE-AIM for health care initiatives in community and clinical settings. Prev Chronic Dis. 2018;15:E02. 29300695 10.5888/pcd15.170271 PMC5757385

[20] Glasgow RE, Vogt TM, Boles SM. Evaluating the public health impact of health promotion interventions: the RE-AIM framework. Am J Public Health. 1999;89(9):1322–7. 10474547 10.2105/ajph.89.9.1322 PMC1508772

[21] National AIDS Control Council (NACC), Ministry of Health - Kenya. Kenya HIV Estimates Report 2020. Available at https://analytics.nsdcc.go.ke/estimates/Kenya-HIV-Estimate-Report-2020.pdf. Accessed 30 Dec 2025.

[22] Primary health care systems (PRIMASYS): case study from Kenya, abridged version. Geneva: World Health Organization; 2017. Licence: CC BY-NC-SA 3.0 IGO.

[23] Ministry of Health, National AIDS & STI Control Program. Guidelines on use of antiretroviral drugs for treating and preventing HIV infection in Kenya 2018 edition. Nairobi, Kenya: NASCOP; 2018.

[24] Ministry of Health, National AIDS & STI Control Program. Kenya HIV prevention and treatment guidelines, 2022 edition. Nairobi, Kenya: NASCOP; 2022.

[25] Creswell JW, Plano Clark VL. Designing and conducting mixed methods research. 3rd ed. Thousand Oaks, CA: SAGE Publications; 2017.

[26] Shelton RC, Chambers DA, Glasgow RE. An extension of RE-AIM to enhance sustainability: addressing dynamic context and promoting health equity over time. Front Public Health. 2020;8:134. 32478025 10.3389/fpubh.2020.00134 PMC7235159

[27] Kohler P, Jiang W, Badia J, Kibugi J, Dyer J, Kadima J, et al. Data-informed Stepped Care (DiSC) to improve adolescent and young adult HIV care outcomes in Kenya: a cluster randomized trial. J Int AIDS Soc. 2025;28 Suppl 3(Suppl 3):e26501. 40622356 10.1002/jia2.26501 PMC12232485

[28] Chhun N, Oketch D, Agot K, Mangale DI, Badia J, Kibugi J, et al. Using FRAME to characterize provider-identified adaptations to a stepped care intervention for adolescents and youth living with HIV in Kenya: a mixed methods approach. J Int AIDS Soc. 2024;227 Suppl 1(Suppl 1):e26261. 10.1002/jia2.26261 PMC11224585 38965971

[29] Chhun N, Mangale DI, Agot K, Owade WA, Kadima J, Badia J, et al. Determinants of implementation of a stepped care intervention for adolescents and youth living with HIV in Kenya: a qualitative evaluation. BMC Health Serv Res. 2025;25(1):702. 10.1186/s12913-025-12875-7 PMC12079965 40369586

[30] Donenberg GR, Merrill KG, Obiezu-Umeh C, Nwaozuru U, Blachman-Demner D, Subramanian S, et al. Harmonizing implementation and outcome data across HIV prevention and care studies in resource-constrained settings. Glob Implement Res Appl. 2022;2(2):166–77. 35411334 10.1007/s43477-022-00042-7 PMC8987520

[31] Day S, Kapogiannis BG, Shah SK, Wilson EC, Ruel TD, Conserve DF, et al. Adolescent participation in HIV research: consortium experience in low and middle-income countries and scoping review. Lancet HIV. 2020;7(12):e844–52. 33275917 10.1016/S2352-3018(20)30269-1 PMC8491773

[32] Tucker JD, Iwelunmor J, Abrams E, Donenberg G, Wilson EC, Blachman-Demner D, et al. Accelerating adolescent HIV research in low-income and middle-income countries: evidence from a research consortium. AIDS. 2021;35(15):2503–11. 34870930 10.1097/QAD.0000000000003049 PMC8901045

[33] Damschroder LJ, Lowery JC. Evaluation of a large-scale weight management program using the consolidated framework for implementation research (CFIR). Implement Sci. 2013;8:51. 23663819 10.1186/1748-5908-8-51 PMC3656778

[34] Malterud K, Siersma VD, Guassora AD. Sample size in qualitative interview studies: guided by information power. Qual Health Res. 2016;26(13):1753–60. 26613970 10.1177/1049732315617444

[35] R Core Team. R: a language and environment for statistical computing. Vienna, Austria: R Foundation for Statistical Computing; 2023. https://www.R-project.org/.

[36] Hsieh HF, Shannon SE. Three approaches to qualitative content analysis. Qual Health Res. 2005;15(9):1277–88. 16204405 10.1177/1049732305276687

[37] Holtrop JS, Rabin BA, Glasgow RE. Qualitative approaches to use of the RE-AIM framework: rationale and methods. BMC Health Serv Res. 2018;18(1):177. 29534729 10.1186/s12913-018-2938-8 PMC5851243

[38] Kohler P, Jiang W, Badia J, Kibugi J, Dyer J, Kadima J, et al. Data Informed Stepped Care (DiSC) to improve HIV care for youth with HIV: a cluster randomized trial. CROI Conference March 3–6, 2024 Poster Presentation. 2024.

[39] Wiltsey Stirman S, Baumann AA, Miller CJ. The FRAME: an expanded framework for reporting adaptations and modifications to evidence-based interventions. Implement Sci. 2019;14(1):58. 31171014 10.1186/s13012-019-0898-y PMC6554895

[40] Weiner BJ, Lewis CC, Stanick C, Powell BJ, Dorsey CN, Clary AS, et al. Psychometric assessment of three newly developed implementation outcome measures. Implement Sci. 2017;12(1):108. 28851459 10.1186/s13012-017-0635-3 PMC5576104

[41] Li H, Shah SK, Healy E, Agot K, Neary J, Wilson K, et al. “[T]he laws need to change to reflect current society”: Insights from stakeholders involved in development, review or implementation of policies about adolescent consent for HIV testing, care and research in Kenya. J Int AIDS Soc. 2023;26(1):e26057. 36642867 10.1002/jia2.26057 PMC9841068

[42] Shah SK, Allison SM, Kapogiannis BG, Black R, Dawson L, Erbelding E. Advancing independent adolescent consent for participation in HIV prevention research. J Med Ethics. 2018;44(7):431–3. 29895555 10.1136/medethics-2018-104959

[43] Shah SK, Essack Z, Byron K, Slack C, Reirden D, van Rooyen H, et al. Adolescent Barriers to HIV Prevention Research: Are Parental Consent Requirements the Biggest Obstacle? J Adolesc Health. 2020;67(4):495–501. 32636140 10.1016/j.jadohealth.2020.05.011 PMC7508889

[44] Mukumbang FC, Beima-Sofie K, Neary J, Li H, Agot K, Healy E, et al. “I feel that I should decide on my own....”: who should be involved in the decision-making process for adolescent involvement in HIV research? BMJ Glob Health. 2023. 10.1136/bmjgh-2023-012966. 37963612 10.1136/bmjgh-2023-012966 PMC10649498

[45] Masquillier C, Wouters E, Mortelmans D, van Wyk B. On the road to HIV/AIDS competence in the household: building a health-enabling environment for people living with HIV/AIDS. Int J Environ Res Public Health. 2015;12(3):3264–92. 25794189 10.3390/ijerph120303264 PMC4377963

[46] Mukumbang FC, Knight L, Masquillier C, Delport A, Sematlane N, Dube LT, et al. Household-focused interventions to enhance the treatment and management of HIV in low- and middle-income countries: a scoping review. BMC Public Health. 2019;19(1):1682. 31842846 10.1186/s12889-019-8020-6 PMC6916449

[47] DeSilva MB, Penwill N, Sabin L, Gifford AL, Li Z, Fujie Z, et al. We don’t dare to tell her … we don’t know where to begin: Disclosure experiences and challenges among adolescents living with HIV and their caregivers in China. Int J Pediatr Adolesc Med. 2018;5(1):5–12. 30805525 10.1016/j.ijpam.2017.11.001 PMC6363272

[48] Simons-Rudolph AP, Iritani BJ, Odongo FS, Rennie S, Gilbertson A, Kwaro D, et al. Adolescent perceptions about participating in HIV-related research studies. Child Youth Serv Rev. 2020. 10.1016/j.childyouth.2020.105262. 32905545 10.1016/j.childyouth.2020.105262 PMC7472997

[49] Alamo ST, Wagner GJ, Ouma J, Sunday P, Marie L, Colebunders R, et al. Strategies for optimizing clinic efficiency in a community-based antiretroviral treatment programme in Uganda. AIDS Behav. 2013;17(1):274–83. 22610422 10.1007/s10461-012-0199-9 PMC3887144

[50] Katongole SP, Mukama SC, Nakawesi J, Bindeeba D, Simons E, Mugisa A, et al. Enhancing HIV treatment and support: a qualitative inquiry into client and healthcare provider perspectives on differential service delivery models in Uganda. AIDS Res Ther. 2024;21(1):47. 39068451 10.1186/s12981-024-00637-0 PMC11282821

[51] Iwelunmor J, Blackstone S, Veira D, Nwaozuru U, Airhihenbuwa C, Munodawafa D, et al. Toward the sustainability of health interventions implemented in sub-Saharan Africa: a systematic review and conceptual framework. Implement Sci. 2016;11:43. 27005280 10.1186/s13012-016-0392-8 PMC4804528

